# Intrauterine negative-pressure therapy (IU-NPT) to treat peritonitis after caesarean section

**DOI:** 10.1515/iss-2020-0014

**Published:** 2020-10-01

**Authors:** Chris-Henrik Wulfert, Christian Theodor Müller, Ahmed Farouk Abdel-Kawi, Wolfgang Schulze, Henning Schmidt-Seithe, Sonko Borstelmann, Gunnar Loske

**Affiliations:** Department of General Surgery, Bundeswehrkrankenhaus Hamburg, Hamburg, Germany; Department for General, Abdominal, Thoracic and Vascular Surgery, Kath. Marienkrankenhaus Hamburg, Hamburg, Germany; Department of Obstetrics, Kath. Marienkrankenhaus Hamburg, Hamburg, Germany

**Keywords:** caesarean section, endoscopy, peritonitis, uterine rupture, vacuum therapy

## Abstract

**Objectives:**

We describe the first application of intrauterine negative-pressure therapy (IU-NPT) for an early rupture of a uterine suture after a third caesarean section with consecutive peritonitis and sepsis. Because all four quadrants were affected by peritonitis, a laparotomy was performed on the 15th day after caesarean section. Abdominal negative-pressure wound therapy (A-NPWT) of the open abdomen was initiated. During the planned relaparotomy, a suture defect of the anterior uterine wall was identified and sutured. In the second relaparotomy, the suture appeared once more insufficient.

**Case presentation:**

For subsequent IU-NPT, we used an open-pore film drainage (OFD) consisting of a drainage tube wrapped in the double-layered film. The OFD was inserted into the uterine cavity via the uterine defect and IU-NPT was established together with A-NPT. With the next relaparotomy, local inflammation and peritonitis had been resolved completely. IU-NPT was continued transvaginally, the uterine defect was sutured, and the abdomen was closed. Vaginal IU-NPT was also discontinued after another eight days.

**Conclusions:**

By using IU-NPT, local infection control of the septic focus was achieved. The infectious uterine secretions were completely evacuated and no longer discharged into the abdominal cavity. As a result of the applied suction, the uterine cavity collapsed around the inlaid OFD. The total duration of IU-NPT was 11 days. The uterine defect was completely closed, and a hysterectomy was avoided. The patient was discharged four days after the end of IU-NPT. IU-NPT follows the same principles as those described for endoscopic negative-pressure wound therapy of the gastrointestinal tract.

This case report describes the first instance of negative-pressure wound therapy (NPWT) being applied in the uterine cavity to manage suture failure with resulting peritonitis and sepsis after a third caesarean section. We were able to establish an organ-preserving procedure for controlling the focus of this rare complication, similar to endoscopic negative-pressure therapy (E-NPT) to treat conditions affecting the gastrointestinal tract.

## Background

NPWT has been used to treat wounds on the body surface that are healing by secondary intention. Negative pressure is applied to perform local debridement, reduce the local oedema and bacterial contamination, to drain wound secretions, promote blood flow and stimulate the growth of granulation tissue [1].

Endoscopic negative-pressure wound therapy (E-NPWT) with open-pore foam or film drainage is an innovative procedure to treat transmural defects of the upper and lower gastrointestinal tract such as anastomotic leaks and perforations. After first applications in the rectum, it was successfully used on the thoracic oesophagus, which is also easily accessible by endoscopy; and later on intraperitoneal bowel sections up to the duodenum as well as the stomach and the cervical oesophagus [2]. A first description of its application to treat urogenital tract conditions involves a bladder injury following rectum extirpation [3].

Worldwide, septic episodes such as endometritis are one of the five most common causes of death associated with childbirth [4]. A caesarean section is an independent risk factor in this context [5]. It is very rare, however, for postpartum peritonitis and sepsis to be caused by caesarean section scar dehiscence. The exact prevalence among postpartum women is unclear but appears to be well below 0.1%. A history of caesarean sections, uterine surgery, trauma or high gestational age increases the risk of wound dehiscence [6], [7], [8].

Abscesses, phlegmons or, because of the possible connection to the abdominal cavity, peritonitis with sepsis can subsequently occur [9]. Depending on the severity of infection, conservative treatment with anti-infective agents may be sufficient, but surgical intervention is often necessary [10]. In addition to laparotomy, hysterectomy may become necessary in the course of surgical treatment as a last resort to control the focal infection [11], [12].

## Case description

### Preoperative history

**Figure 1:**
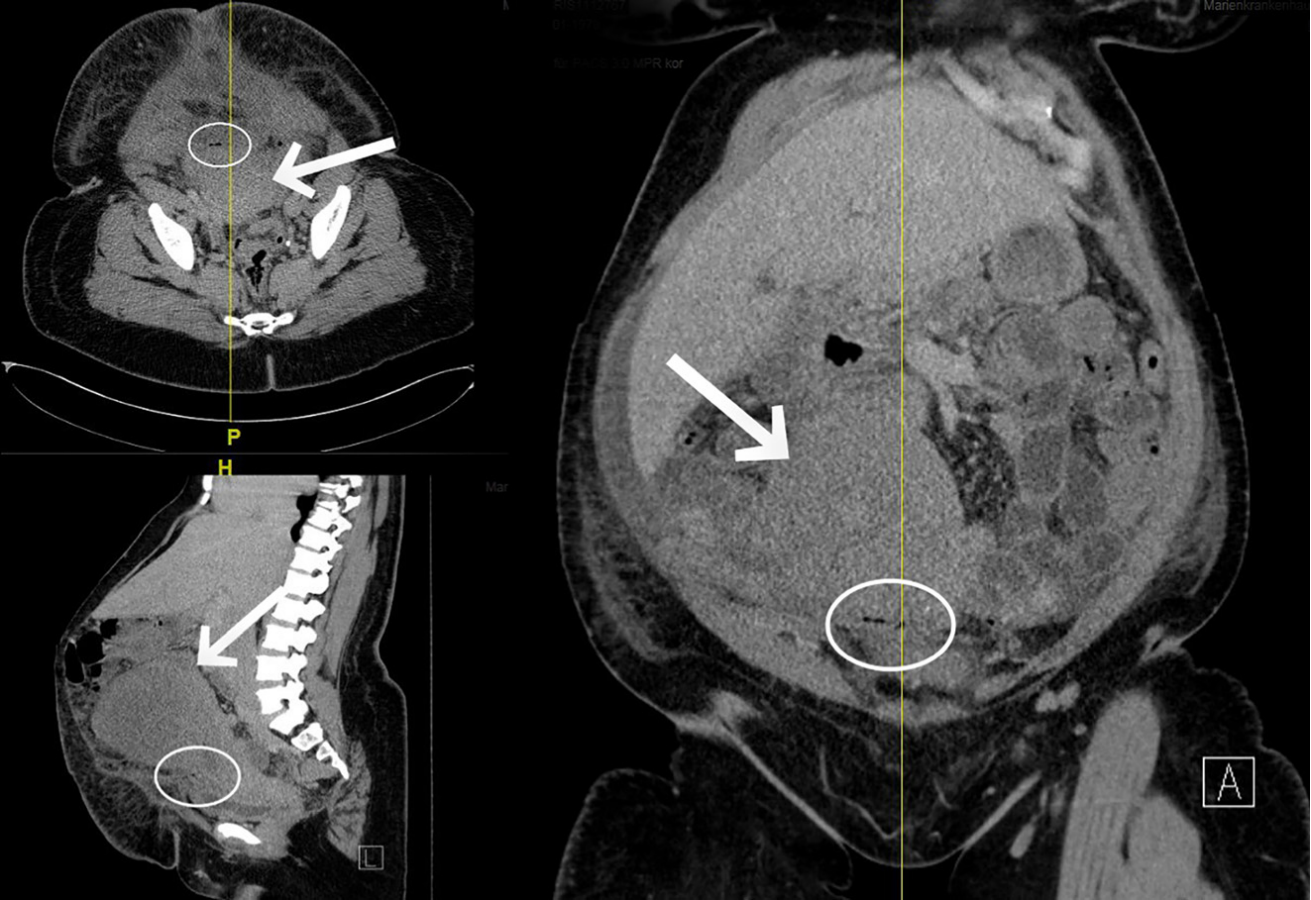
Abdominal CT showing enlarged postpartum uterus (arrows) and few air pockets (circles).

A 39-year-old Black African woman presented to the gynaecological clinic with symptoms of an acute abdomen. Five days earlier, the patient had given birth via caesarean section in another hospital. This was her third caesarean section. At the time of admission, the patient had severe lower abdominal pain with mild signs of peritonitis. Sutures appeared normal. C-reactive protein was elevated at 313 mg/L, while procalcitonin and leucocyte levels were within normal range. An ultrasound examination revealed some free intraperitoneal fluid. The subsequent abdominal CT scan showed a considerably enlarged uterus with postoperative changes and a few air pockets in the area of the incision, as well as diffuse free intraperitoneal fluid (Figure 1). Empiric anti-infective therapy with cefuroxime (3 × 1.5 g) and metronidazole (3 × 500 mg) was initiated.

Multidrug-resistant *Klebsiella pneumoniae* (3-MRGN) was found in a vaginal smear taken from the patient, and the effective spectrum was thus expanded by meropenem (3 × 1 g). After administration of a laxative, the patient’s abdominal symptoms improved, and inflammation markers stagnated. When her condition deteriorated once more, ultrasound-guided paracentesis was performed, providing evidence of *enterococci* and thus the indication for diagnostic laparoscopy on the 15th day after caesarean section.

### Laparotomy and intra-abdominal negative-pressure treatment of peritonitis

A longitudinal laparotomy was performed, as peritonitis was apparent across all four quadrants. Intestinal organ perforation initially could not be confirmed as the cause of the sepsis signs and symptoms. The uterine suture in particular initially appeared to be intact. Following extensive lavage, we initiated intra-abdominal negative-pressure therapy (A-NPT) to treat the patient’s peritonitis. We used an open-pore, double-layered, large-area drainage film (oF) developed specifically for this purpose (Suprasorb® CNP Drainage Film, Lohmann & Rauscher, Germany). The film was spread across the entire abdomen to drain the abdominal cavity completely. The abdominal wall was temporarily adapted with fascial retraction sutures, leaving a fascial gap of 3 cm in diameter. Suction was applied along a polyurethane foam (PU) strip (V.A.C.® GranuFoam™, Kinetic Concepts, Inc., San Antonio, USA) connected to the film, which was guided through the gap from the abdominal to the subcutaneous region. After film sealing, negative pressure (−75 mm Hg) was applied with an electronic vacuum pump (ActiV.A.C, setting: −75 mm Hg, continuous, high intensity; KCI, San Antonio, Texas, USA) [13], [14], [15], [16].

The planned relaparotomy three days later revealed a 3 cm-wide uterine scar dehiscence connected to the abdominal cavity as the septic focus. Just like the vaginal smears taken previously, intra-abdominal smears contained *K. pneumoniae* (3-MRGN). The patient had previously refused a hysterectomy for cultural reasons, which is why, following debridement, the dehiscence was closed with absorbable suture material, using simple interrupted suture technique. A temporary abdominal wall closure was performed. The planned relaparotomy another three days later showed a regression of peritonitis but also a recurrence of the previous suture failure.

### Intrauterine negative-pressure therapy

Drawing on our experience with using E-NPT to close intestinal defects, we decided by interdisciplinary consensus to treat the uterine defect not only from the outside but to also apply intrauterine suction. An intraoperative vaginal examination confirmed a suspected complete cervical stenosis. Drainage from the uterus was therefore obstructed. The first attempt at intraoperative cervical dilatation was unsuccessful. We then performed the first cycle of IU-NPT. Suction was guided directly along the gaping defect in the uterine wall.

**Figure 2:**
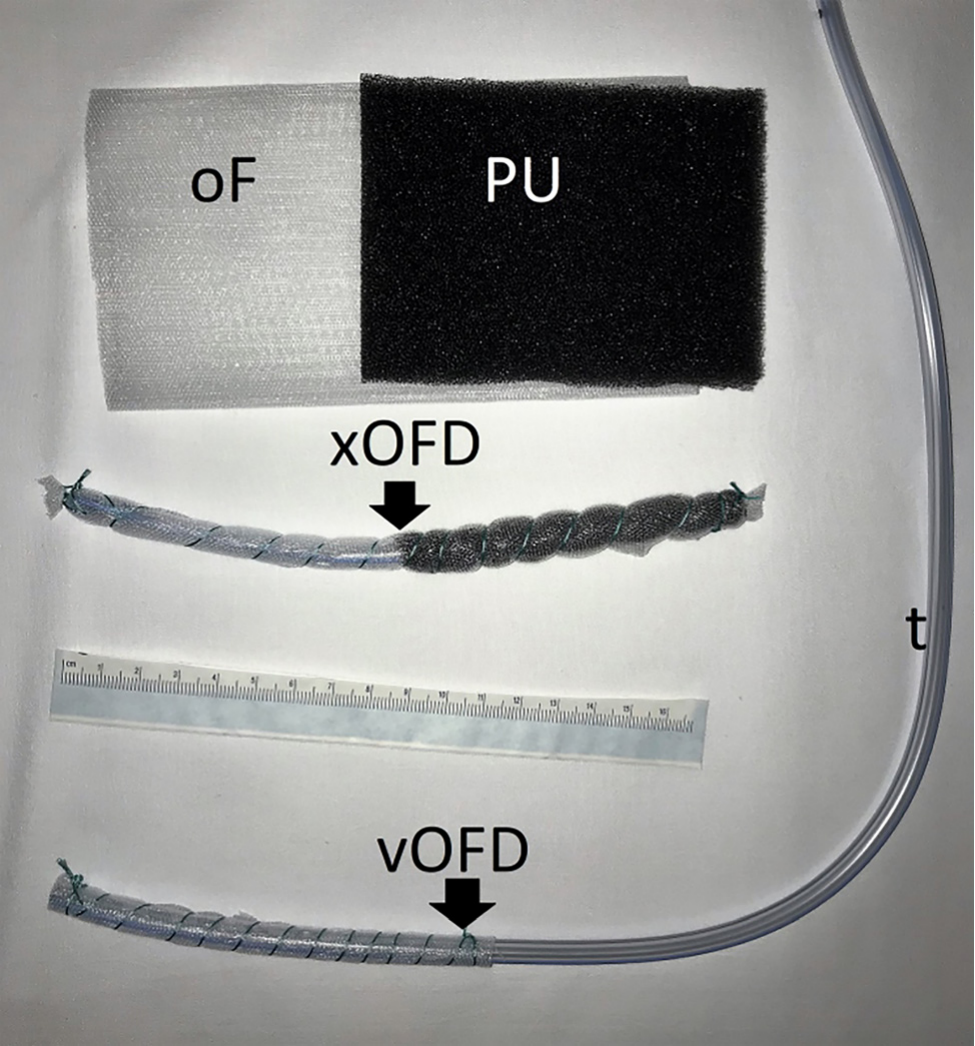
Materials for intrauterine negative-pressure therapy (IU-NPT): double-layered open-pore film (oF), open-pore polyurethane foam (PU), short open-pore drainage element (xOFD) for IU-NPT via the abdominal defect and small-bore vOFD for IU-NPT via the vagina.

**Figure 3:**
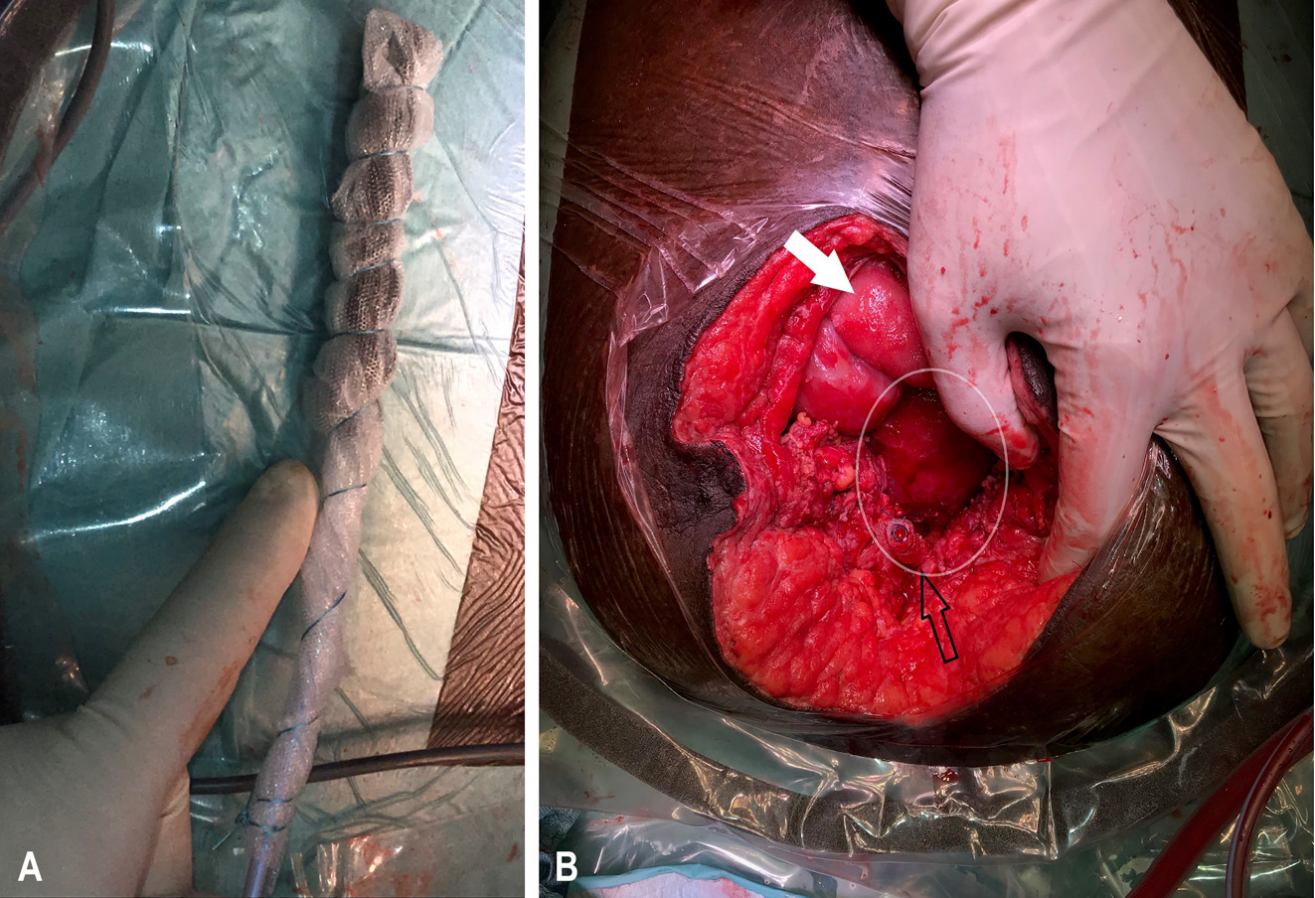
xOFD ex (A) and (B) *in situ*. xOFD (black arrow), small intestine (white arrow), anterior uterine wall (circled in white).

For this purpose, we constructed a single short drainage element (xOFD) with a 20 cm-long distal end of a gastric tube (t) (Ventrol, 18-French, 120 cm, Mallinckrodt Medical, Ireland) with lateral perforations. Half of the xOFD was wrapped with an open-pore PU and then all of it was wrapped with the same thin double-layered drainage film that was used for A-NPT (Figure 2). The half of the xOFD that consisted of film-wrapped PU was inserted into the uterine cavity through the uterine wall defect (Figure 3). The other half of the drainage remained exposed in the lower abdomen. Via this end, the xOFD remained in direct contact with the abdominal drainage film to conduct fluid and negative pressure. The film was connected to an electronic pump (KCI V.A.C.®, KCI USA Inc., San Antonio, Texas, USA, setting: −75 mm Hg, continuous, high intensity). The abdomen was temporarily closed once more with fascial retraction sutures and the foam-film system.

**Figure 4:**
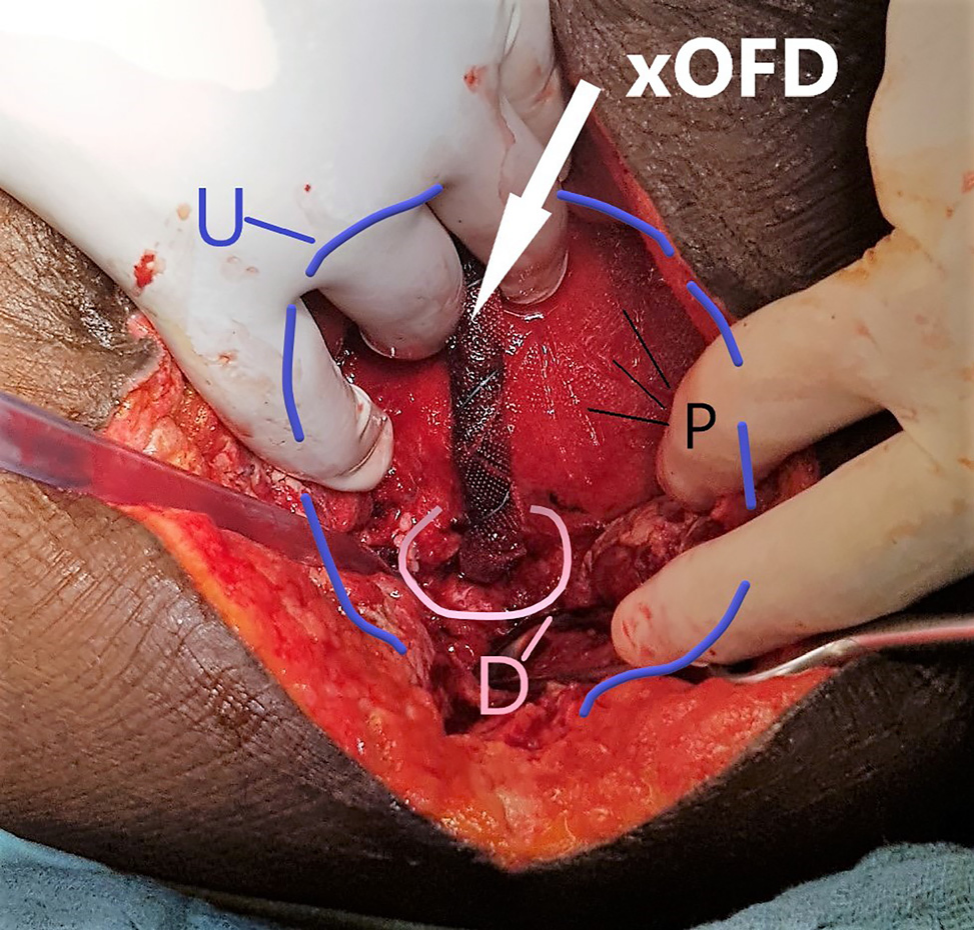
Surgical site after the first intrauterine negative-pressure therapy (IU-NPT) cycle. Half of the xOFD has been inserted into the uterine cavity through the defect (D). Uterus (U), imprint pattern left by the drainage film (P).

The planned relaparotomy after a further four days revealed no remaining signs of peritonitis. The transmission of negative pressure into the uterine cavity by means of the xOFD was sufficiently successful. The xOFD had attached itself to the uterine wall and could be removed with a slight tug (Figure 4). The uterine wound did not show any signs of inflammation. The film left a characteristic imprint where it had been in direct contact with the tissue. Subsequent intraoperative dilation of the cervix uteri with Hegar dilators was successful.

**Figure 5:**
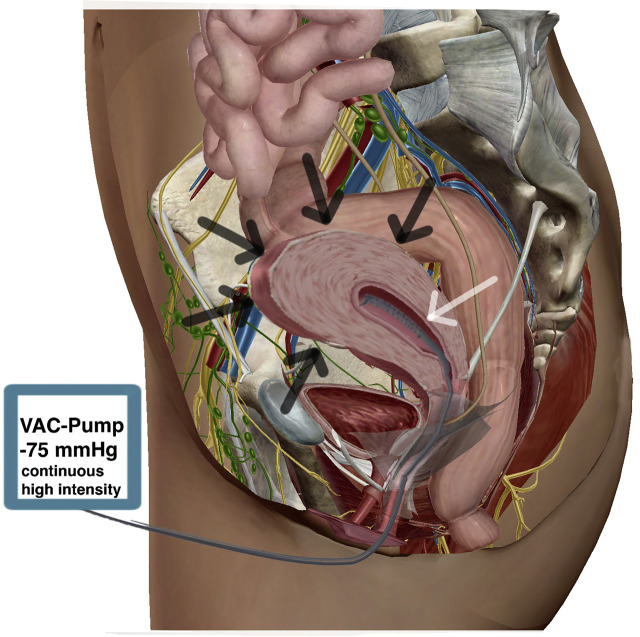
Intrauterine vOFD (white arrow) in contracted uterus (black arrows). Modified with permission of visible body.

We performed the next negative pressure cycle with a vOFD placed via the vagina (Figure 5 and video 00:23 min).

**Video 1:** The video shows the design of the vOFD, the intrauterine placement of the vOFD using the pull-through technique and the intrauterine condition after removal of the vOFD.

This drainage type was constructed by wrapping the distal end of an 18-French gastric tube with a layer of the thin open-pore drainage film over a length of 10 cm. This small-bore drainage type has already been used for various new indications in E-NPT [17]. The vOFD was inserted through the vagina and the previously dilated cervix into the uterine cavity, using the pull-through technique. For this purpose, a soft catheter was first guided through the uterine wall defect and the uterus through the cervix and then out via the vagina. The distal end of the vOFD was then sutured to the tip of the catheter. By pulling on the catheter, we were able to pull the vOFD through to the abdomen (video 1:41 min). The vOFD was disconnected from the catheter and then digitally pushed into the uterine cavity [18]. The uterine defect was closed over the vOFD inside the uterus, using absorbable simple interrupted sutures. We also applied negative pressure (−75 mm Hg) via the vOFD. To prevent accidental displacement, the vOFD was sutured to the vaginal wall. The fascia was definitively closed since signs and symptoms of peritonitis had improved. After adaptation of the wound edges, an external negative pressure film dressing was applied to the skin and subcutaneous connective tissue.

**Figure 6:**
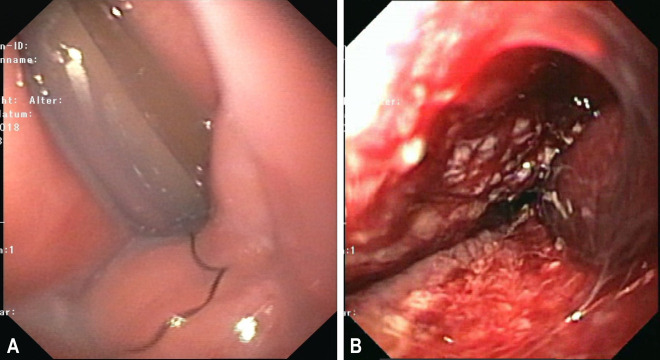
(A) Drainage in the cervical canal and (B) imprint pattern on the uterine tissue left by the film after its removal.

After eight days, the vOFD was removed by simply pulling on the drainage, Therefore the patient was sedated (02:06 min video) and the process was endoscopically monitored. The uterine cavity was then examined with a small endoscope (5 mm in diameter, Olympus N 180). The vOFD had maintained suction over the entire length of the film section in the uterine cavity. The uterine cavity had contracted except for a canal approximately 5–8 mm wide. The characteristic nubby pattern imprint left by the film was apparent on the surface (Figures 2 and 6:36 min video). The suture material used to close the defect was visible from the inside, and the uterine defect was completely adapted. All in all, the wound appeared similar to the findings we know from E-NPT of the gastrointestinal tract.

The total duration of IU-NPT was 11 days. Four days after the end of treatment, the patient was no longer experiencing symptoms and was discharged. An outpatient clinical follow-up examination conducted four weeks after discharge showed normal external wound conditions and no recurrence of symptoms.

## Discussion

This is the first case description of IU-NPT being used to treat a persistent uterine defect after caesarean section in the uterine cavity in combination with abdominal NPT for peritonitis.

Negative pressure applications in the uterine cavity with a large-bore catheter have already been described by Purwosunu et al. The authors used suction to achieve haemostasis in postpartum haemorrhage. The intrauterine vacuum caused the atonic uterus to collapse. This resulted in self-tamponade of the uterus and retonisation of the myometrium. Complete haemostasis was achieved in all 40 women treated with this simple method [13], [15]. In 2019 Haslinger et al. [19] at the University Hospital Zurich have also successfully used negative pressure to treat postpartum haemorrhage on multiple occasions.

In recent years, E-NPT method has been a focus of attention regarding the treatment of oesophageal defects such as anastomotic leaks and perforations. By applying suction to the surrounding tissue, the defect is closed, while contamination is stopped with intraluminal drainage [16]. There are two types of this treatment: intracavitary and intraluminal E-NPT.

In 1926, Martin Kirschner [7], [20], [21], [22] formulated the principles for the treatment of acute suppurative inflammation of the abdomen, stating that “the source of the infection should be eliminated as quickly and nontraumatically as possible.” The essential basic principles involve “blocking the source of the infection, managing and draining the exudate.” These principles continue to apply to the treatment of peritonitis, both for conventional therapy and for treatment with NPT. In our case the contamination of the abdominal cavity was interrupted and drainage of secretion was done at the same time. The surgical principles formulated by M. Kirschner are put into practice with the method of NPT.

This case report demonstrates how the principles that underpin intracorporeal wound healing can also be applied to uterine defects. From a pathophysiological perspective, the failing and necrotically infected uterine suture led to an infection of the abdominal cavity with secretion and bacterial contamination, similar to suture failure in the GI tract. While the exact cause of this instance of uterine suture failure so soon after caesarean section was not definitively established, local hypoperfusion together with bacterial contamination of a local haematoma and subsequent infection leading to necrosis, much like similar occurrences observed in the GI tract, would appear likely. A subsequent abscess can drain into the uterus or through the abdominal wall [2], [7], [23]. However, the higher the uterine suture, the likelier it is that the abdominal cavity will be involved. Our patient also had a complete cervix stenosis, making adequate vaginal drainage of lochia impossible.

When the uterine defect was found, an initial attempt was made to achieve wound closure with sutures while treating the peritonitis with A-NPT [21], [23]. Dedes et al. describe a very similar case in which a uterine defect was treated with NPWT applied externally to the defect. They used PU via the open abdomen in combination with Kerlix Gauze™ and thus achieved closure of the defect after about three weeks of treatment while preserving the organ [7]. A hysterectomy would otherwise have been indicated as a last resort, too [7], [20], [21], [23].

When revision laparotomy once again revealed complete wound dehiscence, we decided to go beyond treating the uterine wound defect from the outside. This time, we performed intracavitary treatment of the uterus, similar to E-NPT of the intestine. This was intended to remove the causative focal infection in the uterus. Since intraoperative dilation of the obstructed cervix, which would have allowed transvaginal drainage, was initially unsuccessful, we performed the first suction cycle along the defect opening, much like the intracavitary variant of E-NPT of the gastrointestinal tract. For this purpose, we used a new type of open-pore drainage, consisting of a piece of drainage tube of about 20 cm in length, completely wrapped in PU foam and open-pore film. Our paper is the first description of this type of drainage. Wrapping the tube with the open-pore drainage material (film and PU foam) made it stiffer, which in turn made it much easier to push it deep into the uterine cavity. For two reasons we decided to wrap the entire tube in the film, rather than just use the PU as an open-pore material. Firstly, the film can be used to create very small-bore drainage systems with a diameter of only a few millimetres [16]. Secondly, the film adheres less firmly to wound beds than PU foam. Negative pressure was applied to this drainage system when it was in direct contact with the drainage film used for abdominal NPWT. Similar approaches based on contact coupling have been described for E-NPWT of the rectum [24]. Suction was conducted into the uterine cavity through contact coupling, enabling sufficient drainage of secretions via the negative pressure system. If early dilatation of the cervix uteri had been possible, we would have opted for initial transvaginal drainage and thus possibly spared the patient another laparotomy. With −75 mm Hg, we used the same level of negative pressure we use for A-NPT. Although there’s a lack of evidence regarding the optimal pressure, concerns for the development of enteroathmospheric fistulas exist due to reduced blood flow in small intestine wall, when negative pressure is applied [25]. Therefore we follow the recommendations of the NPWT expert panel by using continuous NPWT settings of up to 80 mm Hg in the open abdomen [22].

After placement of the vOFD and application of negative pressure for another eight days, the sutured defect healed completely.

We showed that intracavitary NPWT can also be used on the uterus. It is based on the same principles of action as E-NPT of the GI tract. The uterine cavity contracts around the drainage tube, uterine secretions are drained, and the edges of the wound adapt. Similar to E-NPT, additional suturing of the uterine wall may not even be necessary. In cases of uterine scar failure, IU-NPT can be used as an organ-preserving alternative to hysterectomy. Ideally, negative pressure drainage is achieved via the vagina.

Further possible indications include septic conditions of the uterus, where the method can be used after failure of or in addition to conservative treatment. The collapse of the uterus observed during intrauterine therapy could also be a new treatment option for postpartum haemorrhage. The haemostatic contraction of the uterus could be simulated with the application of intracavitary negative pressure [9], [25].

## Conclusion

IU-NPT is an innovative therapeutic approach for the treatment of uterine defects. Both transabdominal and transvaginal treatment are possible with new small-bore drainage types. IU-NPT follows the same principles of action when applied to the uterus as NPT on the gastrointestinal tract. Despite septic conditions, it was possible to perform organ-preserving therapy without hysterectomy. Because of the collapse of the uterus achieved with this treatment, postpartum haemorrhage may be another indication.
