# Supplementary material for: Intrauterine negative-pressure therapy (IU-NPT) to treat peritonitis after caesarean section
Source: Innov Surg Sci. 2020 Oct 1;5(1-2):67–73. doi: 10.1515/iss-2020-0014 (PMC7798308; doi:10.1515/iss-2020-0014)
Supplement: Supplementary file 3 [file iss-5-20200014-s001.pdf]

Reviewer Assessment

Chris-Henrik Wulfert et al.: Intrauterine negative-pressure therapy (IU-NPT) to treat peritonitis after caesarean section

Reviewers' Comments to Original Submission

Reviewer 1: anonymous

Date received: 27-Jun-2020
Reviewer recommendation: **Accept in present form**
Reviewer overall scoring: **Excellent**

Assessment Form scores: 5 = High/Yes; 3 = Medium/Adequate; 1 = Low

Is the subject area appropriate for the journal	5				
Does the title clearly reflect the paper's content?	5				
Does the abstract clearly reflect the paper's content	5				
Do the keywords clearly reflect the paper's content?	5				
Does the introduction present the problem clearly?	5				
Are the results/ conclusions justified?	5				
How comprehensive and up-to-date is the subject matter presented?	5				
How adequate is the data presentation?	5				
Are units and terminology used correctly?	5				
Is the number of cases adequate?	5				
Are the experimental methods/ clinical studies adequate?	5				
Is the length appropriate in relation to the content?	5				
Does the reader get new insights from the article?	5				
Please rate the practical significance.	5				
Please rate the accuracy of methods.	5				
Please rate the statistical evaluation and quality control.	5				
Please rate the appropriateness of the figures and tables.	5				
Please rate the appropriateness of the references.	5				
Please evaluate the writing style and use of language.	5				
Please judge the overall scientific quality of the manuscript.	5				
Are the methods used worthy of reproduction in greater deal?	yes				

Comments to author: The case report describes an innovative technique to treat peritonitis following uterine scar deshiscence after cesarean section. This is a well written paper.

Reviewer 2: anonymous

Date received: 03-Jul-2020
Reviewer recommendation: **Return to author for minor modifications**
Reviewer overall scoring: **Excellent**

Assessment Form scores: 5 = High/Yes; 3 = Medium/Adequate; 1 = Low

Is the subject area appropriate for the journal	5				
Does the title clearly reflect the paper's content?	5				
Does the abstract clearly reflect the paper's content	5				
Do the keywords clearly reflect the paper's content?		4			
Does the introduction present the problem clearly?	5				
Are the results/ conclusions justified?	5				
How comprehensive and up-to-date is the subject matter presented?	5				
How adequate is the data presentation?		4			
Are units and terminology used correctly?	5				
Is the number of cases adequate?	5				
Are the experimental methods/ clinical studies adequate?	5				
Is the length appropriate in relation to the content?	5				
Does the reader get new insights from the article?	5				
Please rate the practical significance.	5				
Please rate the accuracy of methods.	5				
Please rate the statistical evaluation and quality control.	5				
Please rate the appropriateness of the figures and tables.	5				
Please rate the appropriateness of the references.		4			
Please evaluate the writing style and use of language.		4			
Please judge the overall scientific quality of the manuscript.	5				
Are the methods used worthy of reproduction in greater deal?	yes				

Comments to author:

1. In the discussion more details should be given to the comparison between conventional therapy of peritonitis and sepsis and NPT.
2. Differential indications for the different forms of NPT are a bit missing.
3. Why –'75 mm Hg', Rationale ?
4. Mis-Citation : Haslinger is (5), not (6)
5. (6) : incomplete citation
6. The references (2),(3), (6) cannot be found in the text, although they seem to be important.

Authors' Response to Reviewer Comments

Date received: 10-Jul-2020

Response to reviewer 1

Not applicable

Response to reviewer 2

1. In the discussion more details should be given to the comparison between conventional therapy of peritonitis and sepsis and NPT.

In the manuscript, the discussion was supplemented by this note.

"In 1926, Martin Kirschner formulated the principles for the treatment of acute suppurative inflammation of the abdomen, stating that "the source of the infection should be eliminated as quickly and non-traumatically as possible." The essential basic principles involve "blocking the source of the infection and managing and draining the exudate." These principles continue to apply to the treatment of peritonitis, both for conventional therapy and for treatment with negative pressure therapy. In our case the contamination of the abdominal cavity was interrupted and drainage of secretion was done at the same time. The surgical principles formulated by M. Kirschner are put into practice with the method of negative pressure therapy [9,18]."

2. Differential indications for the different forms of NPT are a bit missing.

You are right. The indication for negative pressure therapy is very broad, which is also reflected in the description of our case report. Unfortunately, we can only touch this marginally and have added the information and references to the manuscript in introduction.

"Negative-pressure wound therapy (NPWT) has been used to treat wounds on the body surface that are healing by secondary intention. Negative pressure is applied to perform local debridement, reduce the local oedema and bacterial contamination, to drain wound secretions, promote blood flow and stimulate the growth of granulation tissue[1]."

3. Why –'75 mm Hg', Rationale ?

We thank for the opportunity to address this interesting point. We inserted in discussion part.

"Although there's a lack of evidence regarding the optimal pressure, concerns for the development of enteroathmospheric fistulas exist due to reduced blood flow in small intestine wall, when negative pressure is applied [12]. Therefore we follow the recommendations of the NPWT expert panel by using continuous NPWT settings of up to 80 mmHg in the open abdomen [3]."

4. Mis-Citation : Haslinger is (5), not (6)
5. (6) : incomplete citation
6. The references (2),(3), (6) cannot be found in the text, although they seem to be important.

All References have been revised, corrected and completed.

Reviewers’ Comments to Revised Submission

Reviewer 1: anonymous

Date received: 20-Jul-2020
Reviewer recommendation: **Accept in present form**
Reviewer overall scoring: **Excellent**

Assessment Form scores: 5 = High/Yes; 3 = Medium/Adequate; 1 = Low

Is the subject area appropriate for the journal	5				
Does the title clearly reflect the paper's content?	5				
Does the abstract clearly reflect the paper's content	5				
Do the keywords clearly reflect the paper's content?	5				
Does the introduction present the problem clearly?	5				
Are the results/ conclusions justified?	5				
How comprehensive and up-to-date is the subject matter presented?	5				
How adequate is the data presentation?	5				
Are units and terminology used correctly?	5				
Is the number of cases adequate?					
Are the experimental methods/ clinical studies adequate?	5				
Is the length appropriate in relation to the content?	5				
Does the reader get new insights from the article?	5				
Please rate the practical significance.	5				
Please rate the accuracy of methods.	5				
Please rate the statistical evaluation and quality control.					
Please rate the appropriateness of the figures and tables.					
Please rate the appropriateness of the references.	5				
Please evaluate the writing style and use of language.		4			
Please judge the overall scientific quality of the manuscript.		4			
Are the methods used worthy of reproduction in greater deal?					

Comments to author: See the comments in my last review.

Reviewer 2: anonymous

Date received: 16-Jul-2020
Reviewer recommendation: **Accept in present form**
Reviewer overall scoring: **High**

Assessment Form scores: 5 = High/Yes; 3 = Medium/Adequate; 1 = Low

Is the subject area appropriate for the journal	5				
Does the title clearly reflect the paper's content?	5				
Does the abstract clearly reflect the paper's content	5				

Do the keywords clearly reflect the paper's content?	5				
Does the introduction present the problem clearly?	5				
Are the results/ conclusions justified?		4			
How comprehensive and up-to-date is the subject matter presented?	5				
How adequate is the data presentation?		4			
Are units and terminology used correctly?	5				
Is the number of cases adequate?	5				
Are the experimental methods/ clinical studies adequate?	5				
Is the length appropriate in relation to the content?	5				
Does the reader get new insights from the article?	5				
Please rate the practical significance.	5				
Please rate the accuracy of methods.		4			
Please rate the statistical evaluation and quality control.		4			
Please rate the appropriateness of the figures and tables.		4			
Please rate the appropriateness of the references.	5				
Please evaluate the writing style and use of language.	5				
Please judge the overall scientific quality of the manuscript.	5				
Are the methods used worthy of reproduction in greater deal?					

Comments to author: All questions adequately answered
